# Mental health literacy and stigma among culturally and linguistically diverse adolescents in Australia: a comparative study

**DOI:** 10.1007/s00127-026-03086-4

**Published:** 2026-03-27

**Authors:** Shurong Lu, Laura M. Hart, Anthony F. Jorm, Karen Gregg, Shameran Slewa-Younan, Yang Zhao, Amy J. Morgan

**Affiliations:** 1 Present Address: Melbourne School of Population and Global Health, University of Melbourne, Melbourne, 3010 VIC Australia; 2 Translational Health Research Institute, School of Medicine, Western Sydney University, Sydney, NSW 2751 Australia; 3 Health Services and Systems Research, Duke-NUS Medical School, National University of Singapore, Singapore, 169857 Singapore; 4 The George Institute for Global Health, University of New South Wales, Sydney, 2050 NSW Australia

**Keywords:** Mental health literacy, Stigma, Mental disorder, Adolescents, Culturally and linguistically diverse (CALD), Cross-sectional

## Abstract

**Purpose:**

Using baseline data from a large trial, this study examined general mental health literacy, mental health first aid intentions and actions, and stigmatising attitudes among culturally and linguistically diverse (CALD) adolescents in Victoria, Australia, compared to their non-CALD peers.

**Methods:**

Participants were senior secondary school students from diverse socioeconomic backgrounds. CALD adolescents (*n* = 426) spoke a language other than English at home, while non-CALD adolescents (*n* = 2,714) spoke only English. Outcomes were assessed using two vignettes depicting fictional characters with depression (with suicidal ideation) or anxiety. Multivariate linear or logistic regressions assessed the impact of CALD status on mental health literacy and stigma. *P*-values were adjusted using Holm’s method, where applicable, to mitigate the increased risk of Type I errors due to multiple comparisons.

**Results:**

Adolescents from CALD and non-CALD backgrounds showed similar help-seeking intentions for own mental health and willingness and confidence to assist peers. However, univariate and multivariate analyses consistently indicated that CALD adolescents were much less likely to recognise common mental health problems. Among CALD adolescents, 63.5% recognised depression with suicidal thoughts, compared with 84.2% of their non-CALD peers (odds ratio (OR) = 0.37 [95% CI: 0.21 to 0.63], *p*_adj_ < 0.001); similarly, only 37.3% of CALD adolescents correctly recognised anxiety, compared with 61.8% among non-CALD ones (OR = 0.54 [95% CI: 0.33 to 0.87], *p*_adj_ = 0.036). CALD adolescents also demonstrated significantly lower quality in helpful mental health first aid actions (β = -0.42 [95% CI: -0.70 to -0.14], *p*_adj_ = 0.006) and stronger “weak-not-sick” stigmatising attitudes (β was 0.32 [95% CI: 0.20 to 0.43] for depression and 0.23 [95% CI: 0.10 to 0.35] for anxiety, respectively).

**Conclusion:**

These results suggest significant disparities in multiple components of mental health literacy and stigma among CALD Australian adolescents compared to their non-CALD peers, underscoring the need for the development and implementation of targeted interventions to address these inequities.

**Supplementary Information:**

The online version contains supplementary material available at https://doi.org/10.1007/s00127-026-03086-4.

## Introduction

Adolescence is a critical developmental period marked by increased vulnerability to emotional and behavioural problems [1]. Approximately half of all mental disorders manifest before the age of 18, with peak onset occurring around 14 years of age [2]. Globally, mental disorders are experienced by 14% of young people aged 10–19 years over a 12-month period [3]. In Australia, approximately one in seven adolescents aged 12–17 experience a mental disorder in any given year [4].

Despite the high prevalence of mental disorders during adolescence, many young people do not seek help [5, 6]. In Australia, for example, more than one third (34.9%) of adolescents with emotional or behavioural problems did not use any mental health services in the past year [4]. The reluctance of adolescents to seek formal help for their mental health problems presents a significant barrier to the delivery of appropriate and timely care, and can place them at risk of developing severe or extended mental health problems [7]. Research on help-seeking rates for mental health problems is often aggregated across cultural groups, with limited evidence specifically for CALD adolescents [4]. A recent analysis using electronic medical records found that the cumulative risk of presenting to the Emergency Department with urgent mental health needs was approximately three times higher among CALD youth up to 18 years compared to non-CALD peers (AOR: 2.96, 95% CI = [1.81–4.85]), especially among CALD males [8].

Mental health literacy was first introduced by Jorm and colleagues [9] as the “knowledge and beliefs about mental disorders that aid in their recognition, management, or prevention”. It was later refined to include mental health actions—i.e., actions that individuals or groups take to benefit their own mental health or that of others [10]. When these mental health actions are provided to someone experiencing a mental health problem, a worsening of an existing issue, or a mental health crisis, this is referred to as “mental health first aid” [11].

Since the introduction of the mental health literacy concept, it has garnered significant attention in research as an effective strategy for promoting help-seeking [12]. Mental health literacy is not just about possessing relevant knowledge and favourable attitudes, rather, it involves knowledge and attitudes that are linked to the potential for actions aimed at improving one’s own mental health or that of others [13]. However, much of the existing research on mental health literacy has focused on changing knowledge and beliefs, rather than on altering behaviours to achieve tangible benefits for population mental health. This has led to a growing call for research that examines mental health literacy through the lens of mental health actions [14].

Extensive surveys of mental health literacy have been conducted across various population groups [15, 16], including some among immigrant populations. Studies of adult immigrants in high-income countries such as the USA, Canada, and Australia suggest that individuals from multicultural backgrounds generally have lower levels of mental health literacy and exhibit greater stigma toward people with mental illness [17, 18]. A small number of studies involving multicultural adolescents and young people, e.g., young sub-Saharan African immigrants in Australia, indicate that they often have lower levels of mental health literacy and harbour stigmatising attitudes [19, 20]. Further, recent literature review of 47 studies on immigrant students in high-income countries found that stigma and lack of knowledge about accessing mental health services were common barriers in this population [21].

Australia is a culturally and linguistically diverse (CALD) country, with 22.8% of its population speaking a language other than English at home [22]. The stress of transitioning to a new culture or country can negatively impact mental health, particularly among adolescents, making those from diverse communities more vulnerable to mental health problems than their non-CALD peers [21]. Despite this, there is limited understanding of mental health literacy and stigma among CALD adolescents in Australia, especially as compared to their non-CALD peers [13].

To address this gap, we conducted a cross-sectional, comparative analysis using baseline data from a large randomised controlled trial. This study examined mental health literacy—especially regarding mental health actions—and stigmatising attitudes towards people with mental illness among Australian CALD adolescents, in comparison to their non-CALD peers. Informed by the literature, we hypothesise that CALD adolescents have lower mental health literacy and hold stronger stigmatising attitudes than their non-CALD peers.

## Methods

### Participants

Participants were adolescents aged 14–18 years who completed the baseline online survey for a randomised controlled trial (trial register ID: ACTRN12617000633381) conducted in ten senior secondary schools (6 in metropolitan areas and 4 in regional areas) within the State of Victoria, Australia, during 2017–2021. Once a school agreed to host the trial, all Year 10 students were eligible to participate, unless a parent/guardian opted them out (through passive consent) or the student did not provide their online assent. Students completed the baseline survey during regular class time under the supervision of teaching and research staff. Since the study included information about adolescent mental illness and suicidal thoughts, we asked participants if they felt distressed at the end of the survey. Those who did were offered the opportunity to have a research team member contact them to discuss their feelings. Only a small number reported slight distress, and none required professional support after speaking with team members.

The survey took approximately 20–30 min to complete, and participants were given a AU$5 voucher as a token of appreciation for attending the survey session, regardless of whether they took or completed the survey. Details of participant recruitment and data collection procedure for the trial have been documented elsewhere [23, 24]. Ethics approval for this trial was obtained from the Human Research Ethics Committee at the University of Melbourne (approval ID: 1341238.4) and Victorian Department of Education (approval ID: 2014_002268).

### Measures

The study measured three outcome groups: (1) general mental health literacy (i.e., recognition of common mental health problems, help seeking intention for own mental health, and beliefs about appropriate professional or adult helpfulness); (2) mental health first aid (confidence, intentions, and the quality of intentions or actions); and (3) stigmatising attitudes (i.e., personal stigma and social distance). Outcomes were measured using a vignette-based approach [25], where participants read a vignette describing a hypothetical character with mental illness followed by related questions. All participants were presented with two vignettes that met DSM-5/ICD-10 criteria: the first one depicted a male adolescent (*John*) who was described as experiencing symptoms of a depressive disorder with suicidal ideation, and the second one depicted a female adolescent (*Jeanie*) with symptoms of social anxiety (see Appendix A for a description of the two vignettes).

It is important to note that the study measured both actual mental health first aid actions and a series of mental health first aid intentions (i.e., intentions to take certain actions to help someone with a mental health problem) as the proxy of actions, which were developed based on findings from an expert consensus survey [26]. According to the Theory of Planned Behaviour [27], intentions reflect deliberation about future actions and indicate the effort one is willing to exert if a relevant scenario arises. Previous research has found that adolescents’ mental health first aid intentions effectively predict their practical mental health helping behaviours [24]. Measuring behavioural changes directly in research is challenging, often requiring large sample sizes and extended follow-up periods, which are constrained by limited funding and time for research [12]. Thus, measuring mental health first aid intentions as a practical proxy for actual helping actions offers a promising approach for research aimed at evaluating the effectiveness of mental health initiatives in promoting meaningful helping actions.

### General mental health literacy

#### Recognition of common mental health problems

Immediately following each vignette, participants were asked “*What do you think is wrong with John/Jeanie?*” (i.e., the fictional character in the vignette). Text responses to this open-ended question were coded verbatim in accordance with a structured protocol [28]. To be considered as correct recognition, responses needed to mention either “depression/depressed” or “suicide/suicidal” in response to the John vignette; and any one of “anxiety/anxious”, “social anxiety/ phobia”, or “anxiety disorder” in response to the Jeanie vignette.

#### The quality of intentions to seek help for own mental health problems

This was assessed by the question, “*If I had a problem right now like John/Jeanie’s*,* I would…*”, followed by 10 closed help-seeking items and 1 open item for additional responses. Text from the open item was reviewed verbatim and coded to one of the ten listed items as appropriate or used to generate new items. In this study, no new items were created, so 10 items were included for scoring. Of these items, 5 were recommended (e.g., “*Talk to an adult about it*”) and 5 served as unscored distractors (e.g., “*Improve my diet*”). The five recommended options were scored as ‘1’ if endorsed, and their sum was calculated, with higher scores indicating better quality of self help-seeking intentions. This measure (Appendix B) was adapted from a previously intervention study assessing the intentions of adolescents to help a peer with a mental health problem [29].

#### Beliefs about the helpfulness of adults for the character in the vignette

Participants were asked “*Which of the following people do you think would be helpful*,* harmful*,* or neither helpful nor harmful for John/Jeanie’s problem?*”, followed by 9 options, 6 of which were recommended (e.g., teacher), while 3 served as distractors (e.g., a close friend) (Appendix C). Friends play an important role in youth mental wellbeing; however, adolescents are not equipped to manage their peers’ mental health and evidence emphasises the need for adult involvement [30]. Accordingly, our research encourages adolescents to seek help from trusted adults outside their families (not to diminish family support, which adolescents already commonly access [4]), whilst also supporting their peers. This aligns with the design of evidence-based programs like Teen Mental Health First Aid [29]. The six recommended options were scored as “1” if endorsed as “Helpful”, and their sum was calculated, with higher scores indicating stronger beliefs in adults’ helpfulness for adolescent mental illness.

### Mental health first aid Intended to provide any mental health first aid

Participants were asked, “*If John/Jeanie was a friend*,* I would help him/her*,” with responses ranging from 1 (*Strongly disagree*) to 5 (*Strongly agree*). Responses of “Agree” or “Strongly agree” were coded as “1”, indicating the presence of the intention to help, while other responses were coded as “0”.

#### Confidence to provide any mental health first aid

This was assessed by the question “*If John/Jeanie was a friend*,* how confident would you feel helping him/her?*” with 5 responses from 1 (*Not at all confident*) to 5 (*Extremely confident*).

#### The quality of mental health first aid intentions

This was measured by the previously validated *Mental Health Support Scale for Adolescents* (MHSSA) [23]. The MHSSA asks, “*If John/Jeanie were a friend*,* I would…*,” followed by 12 intention items describing six helpful strategies for responding to the character in the vignette (e.g., “*Listen to John/Jeanie talk about his/her problems*”) and six potentially harmful strategies (e.g., “*Ignore John/Jeanie because s/he is being attention-seeking*”). The response scale ranged from 1 (*Never do this*) to 5 (*Definitely do this*) for each item. The scores for helpful intentions and avoidance of harmful intentions (with harmful items reverse scored) were calculated, with higher scores indicating better quality. For detailed scoring method of the scale, refer to Appendix D for the MHSSA and a previous publication [23].

#### The quality of actual mental health first aid actions

Participants were asked if they had met any peers “*who they thought might have a mental health problem or has experienced a mental health crisis*” within 12 months of the baseline surveys. If participants responded “Yes”, they were then asked if they had offered any help. If “Yes”, they were then asked, “*What did you do to help the person?*” To respond to this question, participants were shown twelve helping actions and asked to respond “Yes” - if the action was taken, or “No” - if not. The 12 helping actions corresponded to the 12 intention items of MHSSA [23] but were expressed as behaviours rather than intentions.

Action items were scored as ‘1’ if a helpful action was taken, or if a harmful action was avoided; otherwise, they were scored as ‘0’. The total scores for helpful actions and harmful action avoidance were calculated separately, with both ranges from 0 to 6, where higher scores indicated better quality of helping actions. Details of this measure are provided in Appendix E.

### Stigmatising attitudes

#### Personal stigma

The Depression Stigma Scale developed by Griffiths et al. [31] was modified and validated for use among young people to measure their levels of personal stigma towards people with mental illness [32]. This scale consists of three distinct dimensions of “Weak-not-sick” (i.e., viewing mental illness as a sign of personal weakness, rather than a medical illness, with 4 items included), “Dangerous/unpredictable” (i.e., seeing people with mental illness as dangerous or unpredictable, with 3 items included) and “Would-not-tell” (i.e., would not tell anyone if they themselves had a mental health problem, with 1 item included). Each dimension was scored as the average of its items and this scale was found to have high internal consistency in adolescents [23].

#### Social distance

The desired social distance (i.e., desired proximity between self and others in social contexts) from a peer experiencing a mental illness was measured by an adapted version of the Social Distance Scale [33]. This scale includes 5 items (e.g., “*Would you be happy to develop a close friendship with John/Jeanie?*”), followed by a 4-point Likert scale ranging from 1 (*Yes definitely*) to 4 (*Definitely not*). The average across items was calculated as the scale score, with higher scores indicating a desire for greater social distance and, consequently, stronger stigmatising attitudes. This scale demonstrated high internal reliability among adolescents for both vignettes [23].

### CALD and non-CALD status

The term CALD is generally considered to refer to people of non-English-speaking background, those born outside Australia, and those whose first language is not English [34]. It recognises differences in religion, spirituality, racial background, ethnicity and language across the Australian population [35]. In this study, students were asked “*Is English your first language? (i.e. the language you speak at home with your family? )*”, followed by options of “Yes” and “No”. Adolescents who self-reported speaking a language other than English at home were defined as CALD, while those who reported English as their first language as non-CALD (Federation of Ethnic Communities Councils of Australia, 2020).

Additionally, data on demographics (i.e., age, gender, school region), personal experiences of mental health problems (Yes/No) and psychological stress levels, measured by the Kessler Psychological Distress Scale (K6), were also collected.

### Statistical analyses

Due to missing responses, 3,092 participants were included in the depression vignette analysis and 3,040 in the social anxiety vignette analysis. Numerical outcome variables were described using means and standard deviations (SD), and categorical variables using frequencies and percentages. The characteristics of groups were compared using *Chi*-square tests for categorical variables and *t*-tests for continuous variables. Differences between groups in components of mental health literacy and stigma were reported using Cohen’s *d*, a standardised effect size that quantifies the difference between group means in standard deviation units, along with their 95% confidence intervals (CIs). Linear regressions were conducted to examine the predictive effects of CALD status on continuous outcomes (i.e., general mental health literacy, mental health first aid intentions and actions, and stigma), adjusting for factors significantly associated with CALD status identified in univariate analyses. Binary logistic regressions were used for the binary outcome (i.e., intended to provide any help to John/Jeanie? Yes/No).

To account for the increased risk of Type I errors due to multiple comparisons, we also calculated the adjusted *p*-values using Holm’s method [36], as applicable, in four separate clusters: repeated t-tests or *Chi*-square tests for univariate analysis, repeated multivariate linear or logistic regressions. The adjusted *p*-values are reported alongside the original *p*-values to provide a clear understanding of the statistical significance after correction. All analyses were conducted using StataSE 19 (College Station, Texas: StataCorp LLC), with a two-sided *p* < 0.05 considered statistically significant.

## Results

### Participants characteristics

As shown in Table 1, the analysis included 3,140 adolescents (mean age 15.90 ± 0.43 years; 48.9% female). Of these, 426 (13.6%) were from CALD backgrounds and 2,714 (86.4%) were from non-CALD backgrounds. Compared to participants in the non-CALD group, those in the CALD group were slightly older (15.97 vs. 15.89 years, *p*_adj_ = 0.003), more likely to attend schools in metropolitan areas (87.6% vs. 65.2%, *p*_adj_ < 0.001), had less experience supporting peers with mental health problems (72.7% vs. 81.6%, *p*_adj_ = 0.008), and were less likely to report having experienced a mental health problems themselves (16.3% vs. 25.8%, *p*_adj_ < 0.001). No statistically significant differences were observed between groups in gender or psychological stress levels.

**Table 1:** Characteristics of participants by CALD status

	All (*n* = 3140)			CALD (*n* = 426)			Non-CALD (*n* = 2174)		Statistic	*p*-value	*p*_adj_^a^
	*N*	Mean (SD)/ %		*N*	Mean (SD)/ %		*N*	Mean (SD)/ %			
**Age** (years, range 14–18)	3,140	15.90 (0.43)		426	15.97 (0.60)		2,714	15.89 (0.39)	*t* = 3.82	**0.001**	**0.003**
Gender											
Male	1549	49.30%		226	53.10%		1,323	48.70%	*F* = 5.21	0.074	0.074
Female	1535	48.90%		189	44.40%		1,346	49.60%			
I identify with another term	56	1.80%		11	2.60%		45	1.70%			
**School region**											
Metro	2,143	68.20%		373	87.60%		1,770	65.20%	*F* = 84.8	**< 0.001**	**< 0.001**
Regional	997	31.80%		53	12.40%		944	34.80%			
Personal experience of supporting peers with mental health problems											
Yes	1,252	80.70%		109	72.70%		1,143	81.60%	*F* = 11.16	**0.004**	**0.008**
No	102	6.60%		9	6.00%		93	6.60%			
Unsure	197	12.70%		32	21.30%		165	11.80%			
Personal experience of having a mental health problem themselves											
Yes	736	24.50%		66	16.30%		670	25.80%	*F* = 21.60	**< 0.001**	**< 0.001**
No	1,572	52.40%		243	60.00%		1,329	51.20%			
Unsure	388	12.90%		62	15.30%		326	12.60%			
I don’t want to answer this question	305	10.20%		34	8.40%		271	10.40%			
Score of psychological stress (range 5–30)	2,990	14.39 (5.87)		405	13.93 (5.60)		2,585	14.46 (5.91)	*t* = 1.70	0.089	0.800

^a^ Adjusted for multiple testing using Holm’s method

### Univariate analysis on associations of CALD status and mental health literacy and stigma

#### Problem recognition

**Fig. 1:**
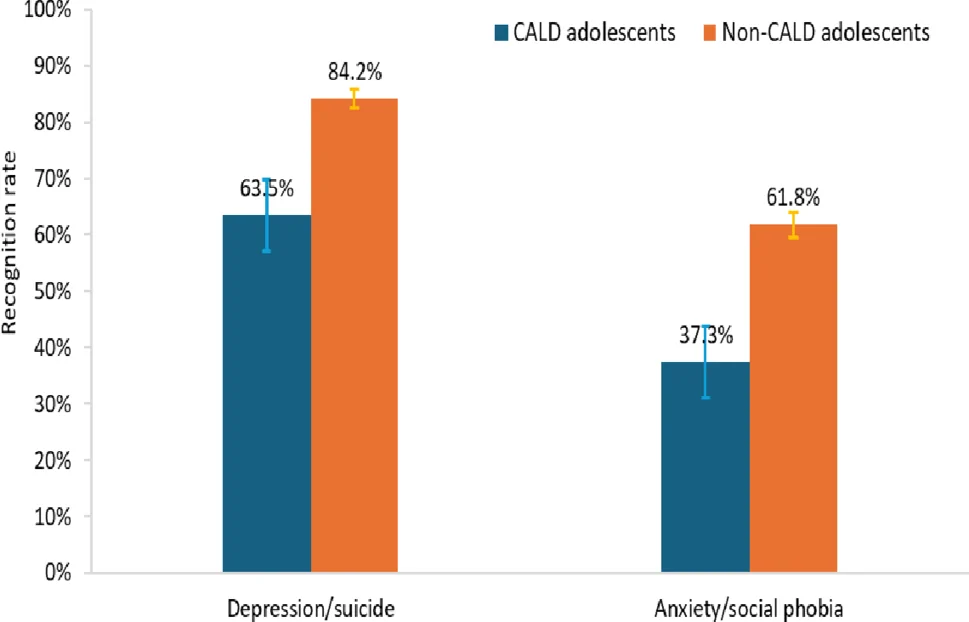
Mental health problem recognition rates among CALD and non-CALD Australian adolescents. ^a^ Bar for 95% CI; The *p*-value and *p*_adj_ < 0.001 for both depression/suicide and anxiety

As presented in Figs. 1 , 63.5% of CALD adolescents correctly identified the mental health problem in the depression/suicide vignette, and 37.3% in the social anxiety vignette. These rates were significantly lower than those of the non-CALD group, within which 84.2% correctly recognised depression/suicide and 61.8% correctly recognised social anxiety (both *p*-value and *p*_adj_ < 0.001 for both vignettes).

#### General mental health literacy, mental health first aid confidence and intentions

The levels of the two other components of general mental health literacy (i.e., help-seeking for own and beliefs in adult helpfulness) as well as mental health first aid confidence and intentions can be seen in Table 2. Compared to non-CALD peers, CALD adolescents scored lower on beliefs about adult helpfulness for John (Cohen’s *d* = -0.12, 95% CI: -0.23 to -0.02), indicating they were less likely to believe adults could help a young person with depression or suicide. However, this difference was not statistically significant for Jeanie in the social anxiety vignette (Cohen’s *d* = -0.06, 95% CI: -0.16 to 0.04).

**Table 2:** Summary of univariate analysis on general mental health literacy, mental health first aid intentions and actions, and stigma among Australian adolescents by vignette and CALD status

	John in the depression vignette				Jeanie in the social anxiety vignette		
	CALD (*N* = 420) Mean (SD) /%	Non-CALD (*N* = 2672) Mean (SD) /%	Cohen’s *d* (95% CI)		CALD (*N* = 409) Mean (SD) /%	Non-CALD (*N* = 2631) Mean (SD) /%	Cohen’s *d* (95% CI)
**General mental health literacy**							
Help seeking for own mental health problem (range 0–5)	2.85 (1.36)	2.96 (1.40)	-0.09 (-0.19 0.02)		2.96 (1.25)	3.05 (1.37)	-0.07 (-0.17 0.03)
Beliefs about adult helpfulness (range 0–6)	3.54 (1.70)	3.74 (1.62)	**-0.12 (-0.23 -0.02)**		3.09 (1.82)	3.21 (1.90)	-0.06 (-0.16 0.04)
**Mental health first aid confidence and intentions**							
Intended to provide any help	88.80%	90.90%	NA		83.10%	86.60%	NA
Confidence to provide any help (range 1–5)	3.78 (0.99)	3.75 (0.96)	0.03 (-0.07 0.14)		3.84 (1.06)	3.82 (0.95)	0.02 (-0.09 0.12)
Intentions to take actions to help (range 0–6)							
* Quality score of helpful intentions*	4.50 (1.37)	4.70 (1.21)	**-0.16 (-0.27 -0.06)**		3.99 (1.57)	4.25 (1.45)	**-0.18 (-0.28 -0.08)**
* Quality score of avoidance of harmful intentions*	3.39 (1.32)	3.72 (1.09)	**-0.29 (-0.40 -0.19)**		3.49 (1.47)	3.82 (1.32)	**-0.25 (-0.35 -0.14)**
**Stigmatising attitudes**							
Personal stigma (range 1–5)							
* Weak-not-sick*	2.30 (0.84)	1.88 (0.73)	**0.57 (0.46 0.67)**		2.33 (0.81)	1.96 (0.78)	**0.47 (0.37 0.58)**
* Dangerous/Unpredictable*	2.41 (0.79)	2.28 (0.75)	**0.17 (0.07 0.28)**		1.99 (0.80)	1.86 (0.78)	**0.17 (0.06 0.27)**
* Not-to-tell*	2.45 (1.16)	2.52 (1.11)	-0.06 (-0.17 0.04)		2.51 (1.06)	2.50 (1.09)	0.02 (-0.09 0.12)
Social distance (range 1–4)	1.97 (0.64)	1.86 (0.62)	**0.17 (0.07 0.27)**		1.76 (0.67)	1.69 (0.65)	**0.11 (0.01 0.21)**

Regarding mental health first aid confidence and intentions, no statistically significant differences were observed between the CALD and non-CALD groups in terms of the percentage of intending to provide any help nor confidence to provide any help. However, CALD adolescents had significantly lower quality scores for both helpful and avoidance of harmful mental health first aid intentions compared to non-CALD adolescents. This pattern was consistent across vignettes.

#### Actual mental health first aid actions towards a peer with a mental health problem

**Fig. 2:**
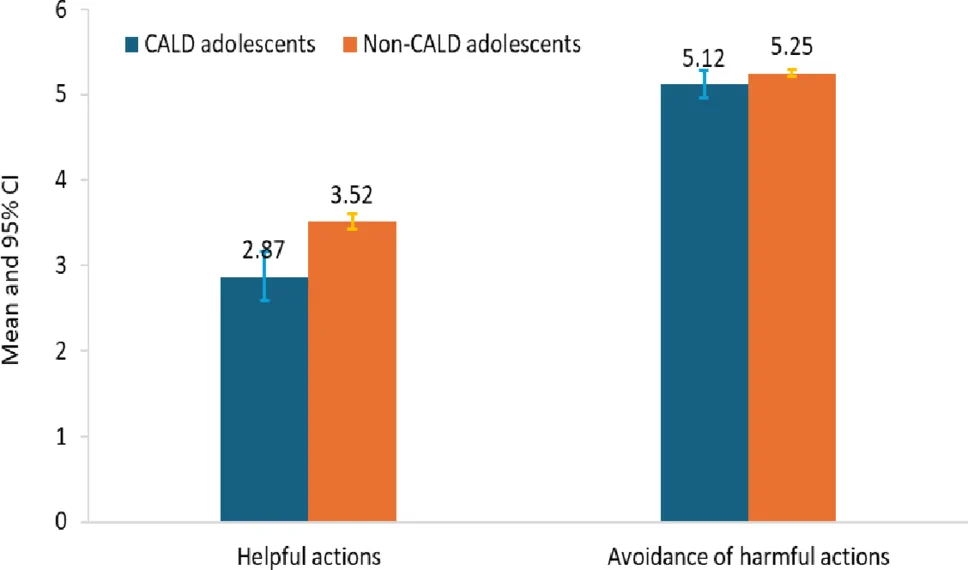
Score of the quality of mental health first aid actions among CALD and non-CALD Australian adolescents. ^a^ Bar for 95% CI; The *p*-value and *p*_adj_ < 0.001 for helpful actions

Among CALD adolescents, 70.9% self-reported helping one or more peers with mental health problems in the 12 months prior to the survey, a significantly lower rate compared to non-CALD adolescents (77.6%, both *p*-value and *p*_adj_ < 0.001). For those who had supported peers with mental health problems, the quality scores of their mental health first aid actions are demonstrated in Fig. 2. The quality score for helpful mental health first aid actions (range 0–6) was 2.87 for the CALD group, significantly lower than the non-CALD group (3.52, Cohen’s *d* = -0.38, 95% CI: -0.56 to -0.21). However, the score for avoidance of harmful actions was similar between the two groups (5.12 for CALD and 5.25 for non-CALD, Cohen’s *d* = -0.16, 95% CI: -0.33 to 0.01).

### Stigmatising attitudes

As demonstrated in Table 2, CALD adolescents exhibited stronger personal stigma than non-CALD adolescents. Specifically, they were more likely to perceive mental health problems related to depression/suicide or social anxiety as a sign of personal weakness rather than a medical condition (i.e., the “Weak-not-sick” dimension, Cohen’s *d* was 0.57 for depression and 0.47 for social anxiety, respectively). They also tended to view individuals with these mental health problems as dangerous or unpredictable (i.e., the “Dangerous/unpredictable” dimension, Cohen’s *d* was 0.17 for both vignettes). Additionally, CALD adolescents expressed a stronger desire for social distancing from individuals with mental health problems, with Cohen’s *d* = 0.17 (95% CI: 0.07 to 0.27) for depression and 0.11 (95% CI: 0.01 to 0.21) for social anxiety.

#### Multivariate linear and logistic regression results for associations of CALD status and mental health literacy and stigma

As summarised in Table 3, multivariate analyses revealed that, after adjusting for potential confounders, including age (continuous), school region, personal experience in supporting peers with mental health problems and own mental health problems, adolescents from CALD backgrounds were less likely to seek help for their own social anxiety problems (β = -0.26, 95% CI: -0.50 to -0.03, *p* = 0.028) and less likely to believe in the helpfulness of adults for depression (β = -0.36, 95% CI: -0.62 to -0.09, *p* = 0.009). CALD adolescents also showed lower quality in avoidance of harmful mental health first aid intentions, evident in both the depression vignette (β = -0.24, 95% CI: -0.41 to -0.06, *p* = 0.007) and the social anxiety vignette (β = -0.25, 95% CI: -0.45 to -0.05, *p* = 0.014). However, these associations were no longer statistically significant when Holm’s adjusted p-values were applied.

**Table 3:** Summary of linear and logistic regressions on the predictive effect of CALD status on mental health literacy and stigma among Australian adolescents by vignette

	John in the depression vignette (*N* = 3092)			Jeanie in the social anxiety vignette (*N* = 3040)		
	β (95% CI)^a^	*p*-value	*p*_adj_^c^	β (95% CI)^a^	*p*-value	*p*_adj_^c^
**General mental health literacy**						
Help seeking for own mental health problem (range 0–5)	-0.18 (-0.42 0.06)	0.137	0.822	-0.26 (-0.50 -0.03)	**0.028**	0.196
Beliefs about adult helpfulness (range 0–6)	-0.36 (-0.62 -0.09)	**0.009**	0.063	-0.02 (-0.34 0.29)	0.882	1.000
**Mental health first aid confidence and intentions**						
Confidence to provide any help (range 1–5)	0.01 (-0.15 0.18)	0.867	0.867	0.00 (-0.16 0.16)	0.997	0.997
Mental health first aid intentions (range 0–6)						
*Quality score of helpful intentions*	-0.12 (-0.30 0.06)	0.198	0.792	-0.08 (-0.29 0.13)	0.455	1.000
*Quality score of avoidance of harmful intentions*	-0.24 (-0.41 -0.06)	**0.007**	0.056	-0.25 (-0.45 -0.05)	**0.014**	0.112
**Stigmatising attitudes**						
Personal stigma (range 1–5)						
*Weak-not-sick*	0.32 (0.20 0.43)	**< 0.001**	**< 0.001**	0.23 (0.10 0.35)	**< 0.001**	**< 0.001**
*Dangerous/Unpredictable*	0.07 (-0.05 0.20)	0.262	0.786	0.02 (-0.10 0.15)	0.745	1.000
*Not-to-tell*	0.06 (-0.13 0.25)	0.562	1.000	0.07 (-0.12 0.28)	0.443	1.000
Social distance (range 1–4)	0.07 (-0.34 0.17)	0.193	0.965	0.02 (-0.08 0.13)	0.672	1.000
	OR (95% CI)^a^	*p*-value	*p*_adj_	OR (95% CI)^a^	*p*-value	*p*_adj_
Intended to provide any help? (“Yes”, “No” as the reference group)	0.78 (0.43 1.42)	0.409	0.818	0.88 (0.53 1.45)	0.61	0.610
Recognition of the mental health problem	0.37 (0.21 0.63)	**< 0.001**	**< 0.001**	0.54 (0.33 0.87)	**0.012**	**0.036**
**Mental health first aid actions towards a peer**^b^ (*n* = 2408, range 0–6)	β (95% CI)^a^			*p*-value	*p*_adj_	
*Quality score of helpful actions*	-0.42 (-0.70 -0.14)			**0.003**	**0.006**	
*Quality score of avoidance of harmful actions*	-0.14 (-0.28 0.01)			0.066	0.066	

^a^ Adjusted for age (continuous), school region, personal experience of supporting peers with mental health problems and own mental health problems

^b^ Across all reported mental health problems

^c^ Adjusted using the Holm’s method, and any *p*_adj_ > 1 were truncated to be 1

Compared to their non-CALD peers, CALD adolescents were 67% less likely to recognise the depression problem (OR = 0.37, 95% CI: 0.21 to 0.63, *p* < 0.001) and 46% less likely to recognise the social anxiety problem (OR = 0.54, 95% CI: 0.33 to 0.87, *p* = 0.012). Regarding the quality of helpful mental health first aid actions towards with peers with any mental health problems, CALD adolescents were 0.42 (95% CI: -0.70 to -0.14, *p* = 0.003) times less than their non-CALD peers. Particularly, these associations were still significant even conservatively tested by the Holm’s adjusted *p*-values.

Multivariate regression analyses also revealed that CALD background was significantly associated with higher personal stigma, particularly in the “Weak-not-sick” belief (β = 0.32 for depression and 0.23 for social anxiety, the *p*-values and *p*_adj_ < 0.001 for both vignettes). However, the associations between CALD status and other dimensions of stigma or social distance weakened and were no longer statistically significant after adjusting for potential confounders.

## Discussion

This study used a cross-sectional, comparative analysis to investigate multiple components of mental health literacy—particularly mental health first aid intentions and actions—and stigma, among a large sample of Australian adolescents (*N* = 3,140). To the best of our knowledge, this study is one of the first of its kind to assess levels of mental health literacy and stigma among adolescents from CALD backgrounds and directly compare these with non-CALD peers from same school settings. The study revealed similarities, as well as significant disparities, in the levels of mental health literacy and stigma among CALD adolescents.

First of all, adolescents from both CALD and non-CALD backgrounds demonstrated similar levels of help-seeking intentions for their own mental health problems, willingness to help peers, and confidence in doing so. According to Ajzen’s Theory of Planned Behaviour [27], these favourable attitudes (i.e., intention, willingness) and perceived behavioural control (i.e., confidence) indicate that CALD adolescents are equally likely to provide or seek help when needed, just like their non-CALD peers.

Nevertheless, CALD adolescents demonstrated poorer quality in mental health first aid intentions compared to their non-CALD peers, particularly in avoiding harmful intentions (e.g., encouraging peers to deal with their problem alone)—a pattern consistently observed in both vignettes (Cohen’s *d* was -0.29 [95% CI: -0.40 to -0.19] for depression and − 0.25 [95% CI: -0.35 to -0.14] for anxiety, respectively). Although these gaps became statistically non-significant with Holm’s adjusted *p*-values, they remained consistently significant in both univariate and regression analyses, even after adjusting for potential confounders. Given the predictive relationship between mental health first aid intentions and actions [24], this suggests that CALD adolescents may be more likely to engage in actions potentially harmful to adolescent mental health (e.g., encouraging peers to handle mental health problems on their own). Poor-quality actions within social networks can delay help-seeking and exacerbate mental illness symptoms, as supported by research on help-seeking behaviour and social support [6]. Therefore, it is necessary, and important, to develop and implement evidence-based interventions, e.g., teen Mental Health First Aid training program [37], to improve the quality of CALD adolescents’ mental health first aid intentions.

CALD adolescents also reported lower beliefs in the helpfulness of adults for addressing adolescent depression (Cohen’s *d* = -0,12, 95%CI: -0.23 to -0.02), an association that remained statistically significant after adjusting for potential confounders though not by the conservative Holm’s adjusted *p*-value. This suggests they are less likely to seek help from trusted adults when supporting peers with depression problems, consistent with previous research reporting low help-seeking rates among Latinx youth [5]. Without timely engagement and support from adults (e.g., parents, teachers, or school counsellors), this reluctance can significantly hinder access to health services for adolescents [38]. Family support, including open communication, emotional support, and guidance, is critical for adolescents’ mental health, particularly for those from CALD backgrounds [39]. However, a large-scale study across 30 countries reported that immigrant adolescents report lower levels of family support compared to their non-immigrant peers [40]. Hence, identifying and implementing best practices to empower adults living with adolescents, such as parents, to support those with mental health problems might serve as a potential pathway to promote help-seeking among CALD adolescents.

The robust association between CALD status and stigmatising attitudes, such as perceiving individuals with mental health problems as “weak” rather than medically ill (Cohen’s *d* of CALD status was 0.57 [95% CI: 0.46 to 0.67] for depression and 0.47 [95% CI: 0.37 to 0.58] for anxiety, respectively), aligns with findings from other cross-cultural research [41]. While misconceptions and stigma surrounding mental illness are universal, their expression varies across cultural contexts and value frameworks [42]. Cultural beliefs about the causes and risks of mental illness shape attitudes and responses to individuals experiencing mental health challenges [43]. In collectivistic societies, such as those with Chinese cultural values, mental illness is often perceived as a source of familial shame [44]; in some African communities, beliefs linking mental illness to demonic or spiritual possession can evoke fear-based emotional responses [19]. Addressing disparities in stigma and mental health literacy among CALD groups requires a comprehensive understanding of cultural norms and beliefs [13]. Future interventions should be designed with these factors in mind to ensure their cultural relevance and effectiveness.

This study has several strengths including its large sample size (> 3,000) and the inclusion of both CALD and non-CALD adolescents from same school settings [23, 24], enabling a reliable assessment of outcomes. Additionally, the study employed a comprehensive measurement of mental health literacy, with a particular emphasis on mental health actions—an area that has been underexplored. Notably, it is among the first to use the novel measure—MHSSA—to evaluate the quality of mental health first aid intentions and the quality of actual mental health actions among adolescents, introducing a valuable approach for future research in this field.

However, the findings should be interpreted with some limitations. First, using self-reported language spoken at home as the criterion for defining CALD status may have introduced misclassification. The reported percentage of CALD students in this study (13.6%) was notably lower than the national figure, which states that approximately 25% of primary and secondary school students learn English as an additional language or dialect in Australia [45]. In English-speaking countries, it is not only whether English is spoken at home, but also the extent to which it is spoken that also matters. For example, for children of parents with mixed cultural backgrounds, the language used to communicate with children may be different across parents and thus children may use both English and another language. Consequently, in our study, participants who spoke another language at home, but who primarily used English, may have listed English as their “first language”, leading to their classification as non-CALD. Second, the measures used in this study to assess intentions and actions [23] and stigma [32, 33] were primarily validated for English-speaking populations, with limited evidence for their reliability, validity, and cultural appropriateness for CALD groups. Therefore, we cannot discount the possibility that these measurement limitations played a part in the observed findings. Third, despite our comprehensive measurement of relevant variables, residual confounding may exist in the multivariable regression results. We recognise that socioeconomic status can be linked to the presentation of mental disorders and attitudes towards individuals with mental illness [46], however, we could not adjust for socioeconomic status in our analyses because assessing it through adolescent self-report is problematic; adolescents typically lack the perspective and experience to understand their family’s objective financial circumstances. Lastly, future research with larger samples of CALD adolescents is necessary to verify some inconclusive findings from this study, which became statistically non-significant when more conservatively estimated using Holm’s adjusted *p*-values.

## Conclusion

This study reveals that adolescents from CALD backgrounds have similar help-seeking intentions for their own mental health, as well as a willingness and confidence to assist peers with mental health problems. However, they are less likely than their non-CALD peers to recognise common mental health problems, such as depression, suicide, or anxiety. They also exhibit lower-quality in helpful mental health first aid actions and hold more stigmatising attitudes towards individuals with mental illness. These disparities underscore the need for future research to develop and implement targeted interventions to address these inequities.

## Supplementary Information

Below is the link to the electronic supplementary material.

Supplementary Material 1

## Data Availability

Raw data of this study cannot be shared due to ethical restrictions in accordance with the approved research protocol from the University of Melbourne and Victorian Department of Education under which the study was conducted, but de-identified data are available from the corresponding author on reasonable request.

## Abbreviations

CALD: Culturally And Linguistically Diverse
MHSSA: The Mental Health Support Scale for Adolescents
CIs: Confidence Intervals
