# Supplementary material for: Mental health literacy and stigma among culturally and linguistically diverse adolescents in Australia: a comparative study
Source: Soc Psychiatry Psychiatr Epidemiol. 2026 Mar 27;61(9):1715–26. doi: 10.1007/s00127-026-03086-4 (PMC13578041; doi:10.1007/s00127-026-03086-4)
Supplement: Supplementary file 1 [file 127_2026_3086_MOESM1_ESM.docx]

**Appendices**

# **A. The two vignettes used in the study**

## **John's Story (Depression/suicide vignette)**

John is a 16 year old who has been unusually sad and miserable for the last few weeks. He is tired all the time and has trouble sleeping at night. John doesn’t feel like eating and has lost weight. He can’t keep his mind on his studies and his marks have dropped. He puts off making any decisions and even day-to-day tasks seem too much for him. His parents and friends are very concerned about him. John says he will never be happy again and believes his family would be better off without him. John says he feels so desperate, he has been thinking of ways to end his life.

## **Jeanie’s story (Anxiety/social phobia vignette)**

Jeanie is a 16 year old living at home with her parents. Jeanie started at your school last year and you are the only friend she has made so far. She seems very shy and when you ask her why she doesn't make more of an effort, she says she would really like to make more friends but is scared that she’ll do or say something embarrassing when she’s around others. Although Jeanie’s schoolwork is OK she rarely says a word in class and becomes incredibly nervous, trembles, blushes and seems like she might vomit if she has to answer a question or speak in front of the class. At her house you have seen that Jeanie is quite talkative with her family, but becomes quiet if anyone she doesn’t know well comes over. She has stopped answering the phone and doesn't come to parties anymore. Jeanie says she knows her fears are unreasonable but she can’t seem to control them and this really upsets her.

# **B. Survey question and response options for measuring the quality of intentions to seek help for own mental health problems**

**If I had a problem right now like John’s, I would…**

*Please note: you can select more than one option.*

1. Try to deal with it on my own.
2. Talk to a friend about it.
3. Talk to a health professional about it (e.g. a counsellor, GP or psychologist).
4. Talk to an adult (other than a health professional) about it (e.g. a parent or teacher).
5. Improve my diet.
6. Try to get more sleep.
7. Spend more time with friends
8. Try to do more exercise.
9. Look for information about my problem on the internet
10. Do nothing
11. Other (please specify):______________________________________________

**Scoring instructions**

Response options: Yes (1)/No (0)

Scoring: sum items 2, 3, 4, 7, 9. Total scores can range from 0-5, with higher scores indicating better quality of self help-seeking intentions.

# **C. Survey question and response options for measuring beliefs about the helpfulness of adults for the character in the vignette**

**Which of the following people do you think would be helpful, harmful, or neither helpful nor harmful, for John/Jeanie’s problem?**

1. Close friend
2. Counsellor
3. Family member
4. General practitioner (GP) or family doctor
5. Minister or priest
6. Parent
7. Psychologist
8. School Welfare Coordinator/ School Counsellor
9. Teacher

**Scoring instructions**

Response options: Helpful (1), Neither helpful nor harmful (2), Harmful (3)

Scoring: sum items 2, 4, 5, 7, 8, 9. Total scores can range from 0-6, with higher scores indicating stronger beliefs in adults’ helpfulness for adolescent mental illness.

# **D. Items of the Mental Health Support Scale for Adolescents (MHSSA)**

*If John/Jeanie were a friend, I would:*^1^

Tell John/Jeanie what s/he needs to do to fix his problems (harmful)^2^

Invite John/Jeanie to hang out and do something fun with me (helpful)^2^

Ignore John/Jeanie because s/he is being attention-seeking (harmful)

Let John/Jeanie know I won’t want to be friends with him any more if s/he’s like this all the time (harmful)

Avoid talking about suicide because it might put the idea in John/Jeanie’s head (harmful)

Tell John/Jeanie I have noticed something seems wrong and I want to make sure s/he is okay. (helpful)

Suggest John/Jeanie tell a health professional about his/her problems (e.g. a counsellor, GP or psychologist). (helpful)

Suggest John/Jeanie tell an adult (other than a health professional) about his/her problems (e.g. parent or teacher). (helpful)

Ask John/Jeanie if s/he is thinking of suicide. (helpful)

Encourage John/Jeanie to take responsibility and deal with his/her problems on his/her own. (harmful)

Listen to John/Jeanie talk about his/her problems. (helpful)

Not do anything. (harmful)

^1^ Response scale: 1 Never do this, 2 Unlikely to do this, 3 Not sure, 4 Probably do this, 5 Definitely do this; An extra item of “*Other (please specify)*” was used as the 13^th^ item in the trial, but was not included for analysis in this study, so it is not presented here.

^2^ The notes of (harmful)/(helpful) were not part of the scale, but used here to demonstrate the property of different items.

# **E. Survey questions related to adolescents’ first aid actions**

In the last 12/18 months have you *had contact with anyone about your age* *(i.e. between 14-18 years old)* who you thought might have a mental health problem or has experienced a mental health crisis? *Please do not report any adults that you have had contact with. For this question, we are just interested in people around your age who you may have helped.*

- Yes – go to next question
- No – skip to next section
- Not sure – go to next question
- I don’t want to answer this question – skips to next section

Please tell us *how many people about your age* you had contact with who you thought might have a mental health problem or experienced a mental health crisis:

- 0 – skip to next section
- 1 – go to next question
- 2 – go to next question
- 3 – go to next question
- More than 3 (please specify) – go to next question

If you had contact with more than one person about your age who you thought might have a mental health problem or experienced a mental health crisis, please answer the following questions ***about the person who you know best***. If you only had contact with one person, please answer the following questions about that person. What was the mental health problem or mental health crisis?

Did you offer the person any help?

- Yes – go to next question
- No – skip to next section
- Not sure – go to next question

*What did you do to help the person?*

|  | Yes | No |
| --- | --- | --- |
| Tell John/Jeanie what s/he needs to do to fix his problems (harmful) |  |  |
| Invite John/Jeanie to hang out and do something fun with me (helpful) |  |  |
| Ignore John/Jeanie because s/he is being attention-seeking (harmful) |  |  |
| Let John/Jeanie know I won’t want to be friends with him any more if s/he’s like this all the time (harmful) |  |  |
| Avoid talking about suicide because it might put the idea in John/Jeanie’s head (harmful) |  |  |
| Tell John/Jeanie I have noticed something seems wrong and I want to make sure s/he is okay. (helpful) |  |  |
| Suggest John/Jeanie tell a health professional about his/her problems (e.g. a counsellor, GP or psychologist). (helpful) |  |  |
| Suggest John/Jeanie tell an adult (other than a health professional) about his/her problems (e.g. parent or teacher). (helpful) |  |  |
| Ask John/Jeanie if s/he is thinking of suicide. (helpful) |  |  |
| Encourage John/Jeanie to take responsibility and deal with his/her problems on his/her own. (harmful) |  |  |
| Listen to John/Jeanie talk about his/her problems. (helpful) |  |  |
| Not do anything. (harmful) |  |  |
